# Storage stability of organophosphorus pesticide residues in peanut and soya bean extracted solutions

**DOI:** 10.1098/rsos.180757

**Published:** 2018-07-25

**Authors:** Gang Guo, Naiwen Jiang, Fengmao Liu, Yanli Bian

**Affiliations:** College of Science, China Agricultural University, Beijing 100193, People's Republic of China

**Keywords:** organophosphorus pesticide, storage stability, solvent, matrix, light, temperature

## Abstract

This study was performed to determine the storage stability of organophosphorus pesticide residues in high oil content commodity matrices, peanut and soya bean. The storage conditions included different types of solvents (ethyl acetate, acetone and hexane) and corresponding extracted matrix solutions, light and temperature. It was found that three pesticides degraded quickly especially in ethyl acetate solvent. They decreased greater than 30% when stored for 3 days at −20°C in ethyl acetate; the results showed that the stability could be improved in the extracted matrix solutions. Light had a slight effect for stability of phorate and fenthion, while it played an important effect for disulfoton with the exception of ethyl acetate as solvent. Even at −20°C, exposure to solvents or extracted matrix solution resulted in 40.67, 96.33 and 35.07% loss of phorate, disulfoton and fenthion. Hence, it could be assumed that these three residues could be more stable at lower temperature, in the dark and in acetone or hexane extracted peanut and soya bean solutions.

## 1. Introduction

The evaluation of pesticide residues in food stuffs is currently an indispensable means of ensuring food quality and protecting consumers against potential health risks. Assessment of the stability of the different analytes in a matrix was a preliminary requirement to determine the acceptable conditions and terms of storage for screening purposes [1,2] (http://www.fao.org/fileadmin/templates/agphome/documents/Pests_Pesticides/JMPR/Report12/Dichlorvos.pdf; http://www.fao.org/fileadmin/templates/agphome/documents/Pests_Pesticides/JMPR/Reports_1991-2006/Report1993.pdf ). Some pesticides are very unstable in matrices and organic solvents [3,4]. In most cases, sample transportation from field to laboratory can take some time, then sample preparation takes place after storage for a period of time [5]. Sample preparation is considered as one of the most important procedures in pesticide residue analytical methods [6]. There is an increasing demand for development of improved analytical methods for the determination of pesticide residues in matrices [7,8]. Few researchers focus on the period from sample preparation to injection. One of the requests for laboratory accreditation systems should include stability studies during development or validation of analytical methods. The design of stability studies was described in regulatory documents [2] (http://www.fao.org/fileadmin/templates/agphome/documents/Pests_Pesticides/JMPR/Reports_1991-2006/Report1993.pdf) and in the literature [9–13]. The criteria of OECD 509 for stability were established and a loss of 30% was already stated as the limit of stability for standard solutions [12,13]. Suitable limits to be applied in terms of acceptability criteria for fortified control samples were completely dependent on the repeatability of the analytical method and could be related to the objectives of the analytical method (screening or confirmatory purposes). Regarding research on the stability in pure solvent, Bernal & Jiménez [14] studied the stability of coumaphos, bromopropylate, amitraz and chlordimeform in methanol and n-hexane, and breakdown products were also detected. They found that temperature, light and solvent types affected stability of the above pesticides. Maštovská & Lehotay [1] evaluated the stability of 31 kinds of pesticides in ethyl acetate, acetonitrile, acetone, n-hexane, isooctane and toluene. Degradation of captan, folpet and dichlofluanid in acetonitrile was observed only in certain lots of the tested acetonitrile, but the stability of these analytes could be dramatically improved by the addition of 0.1% (v/v) acetic acid. Dicofol and chlorothalonil were unstable in acetone, and pesticides with a thioether group (e.g. fenthion, disulfoton) also degraded in the test. With respect to pesticide stability in extracts, Vladimir & Jan [15] observed that chlorothalonil and iprodione degraded significantly in cabbage ethyl acetate extracts. Phosphate ester pesticides iprodione and pirimicarb degraded rapidly after storage for 40 days at 20°C. The selected pesticides were more stable in wheat extract than in orange and cabbage extracts. Kobayashi *et al.* [16] investigated the stability of triflumizole and its metabolic product FM-6-1 in chilli and lily extracts. It was observed that the stability was related to chlorophyll content, temperature and light. A large number of studies have shown that there are many factors affecting the storage stability, especially, the influence of temperature on the stability of pesticide residue samples in extracting solvents.

We therefore analysed the effect of different types of solvents (ethyl acetate, acetone and hexane), as well as peanut and soya bean extracted solutions on storage stability of three organophosphorus pesticides (phorate, disulfoton and fenthion), using a multi-residue screening method based on GC-FPD, and also investigated the effect of light and temperature. This study gave the opportunity to determine suitable conditions for dissolving certain pesticide standards using different pure solvents.

## 2. Experimental

### 2.1. Reagents and materials

The standards of phorate (98.7%), disulfoton (98.5%) and fenthion (97.0%) were purchased from the Agricultural Environmental Protection Institution of Tianjin (Tianjin, China). The analytical reagents acetonitrile, acetone, ethyl acetate and hexane were obtained from Burdick & Jackson. Ultra-pure water was purified on a Milli-Q water purification system (Millipore, Bedford, MA, USA).

A mixture stock solution containing the above-mentioned standards at 1000 mg l^−1^ was prepared in acetonitrile. The stock solution was stored at 4°C. Working standard solutions were prepared daily by diluting the stock solution with acetonitrile to the required concentrations.

Peanut and soya bean samples were purchased from the local market.

### 2.2. Instrumentation

**Figure 1.**
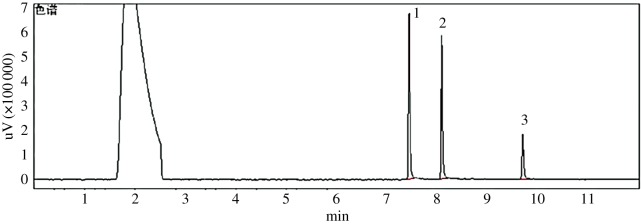
The chromatogram of the three organophosphorus pesticides. 1, phorate; 2, disulfoton; 3, fenthion.

A Shimadzu GC-2010 series gas chromatography system (Shimadzu, Japan) consisting of flame photometric detector (FPD), an automatic sample injector and Shimadzu Chem-Station was used for analysis. An RTX-17 column (30 m × 250 µm × 0.25 µm, Shimadzu) was used for the separation of these pesticides. The injector and detector were operated at 200°C and 260°C, respectively. Operating conditions were as follows: initial temperature was 120°C and held for 1 min, then increased to 260°C at 20°C min^−1^ and held for 4 min. Injections were carried out in the splitless mode (electronic pressure control), and the injection volume was 1 µl. Nitrogen (purity 99.999%) was used as the carrier at a constant flow rate of 1 ml min^−1^, the flow of H_2_ and air was 80 and 110 ml min^−1^, respectively. The chromatogram of the organophosphorus pesticides is shown in figure 1.

### 2.3. Sample preparation

Samples (5 g) of peanut or soya bean were put into a sealed plastic cylindrical container (50 ml) with 10 ml deionized water. After standing for 10 min, the samples were extracted with 20 ml acetonitrile in the oscillator for 30 min and then centrifuged for 5 min at 3800 r.p.m. Supernatant (15 ml) was transferred into another tube, 10 ml acetonitrile was added, and the extraction process was repeated. NaCl (10 g) was added to 5 ml supernatant and the mixture was immediately hand-shaken for 1 min and centrifuged at 3800 r.p.m. for 5 min. The mixture was stored at −18°C for 10 h, then an aliquot of the acetonitrile layer (6 ml) was evaporated to dryness under vacuum at 40°C, then dissolved in 1 ml certain solvents (n-hexane, acetone and ethyl acetate, respectively) and purified with 30 mg C18. Finally, 0.5 ml purified supernatant was passed through a 0.22 µm filter membrane and for the injection into the gas chromatography FPD.

### 2.4. Storage stability experiments

The samples prepared in §2.3 were used to investigate the storage stability of pesticides over a period of 7 days at 35°C and 3 days at 4°C or −20°C in clear vial or brown vials, respectively. The stability of residues in the extracted solutions and in pure solvent was compared. The spiked concentrations were 1 mg kg^−1^. The guidelines of OECD regarded field trials samples and fortified samples were both permitted to be used for research on the storage stability. Specifically, if the remaining pesticide residue was equal to or greater than 70%, it was defined as stable, namely that the storage conditions of that pesticide in that matrix was stable, and the stable period was its corresponding days. In this study, the recovery of these pesticide residues was used to indicate the remaining residue. The calculation of recovery was the ratio of residual level stored for a period of time compared with initial residue.

## 3. Results and discussion

### 3.1. Method validation

Identification and quantification of phorate, disulfoton and fenthion was based on the GC retention time, and the peak areas were compared against the standard calibration curves. The limits of detection (LODs) and the limits of quantitation (LOQs) for phorate, disulfoton and fenthion were considered to be the concentrations produced at a signal-to-noise (S/N) ratio of 3 and 10, respectively. Further, the data for LOD and LOQ were retained to the first place after the decimal point according to the round-off principle. In this study, the LOQs for the three pesticides were 0.05, 0.1 and 0.1 mg kg^−1^, respectively.

The samples were prepared using a modified QuEChERS method. The accuracy was evaluated by determining the spiked recoveries at various levels in peanut and soya bean. The recoveries of phorate, disulfoton and fenthion in the peanut and soya bean were within 89.3%–94.5% at the concentration of 1.0 mg kg^−1^. The results are shown in table 1.

**Table 1.** The recoveries of pesticides in the soya bean and peanut (*n* = 3).

	soya bean		peanut	
pesticide	recovery (%)	RSD (%)	recovery (%)	RSD (%)
phorate	89.32	1.84	92.88	3.58
disulfoton	91.67	1.01	92.01	4.13
fenthion	89.81	3.35	94.46	7.68

### 3.2. Factors influencing storage stability

#### 3.2.1. Solvents and matrices

The stability of these three organophosphorus pesticides in matrix solution which was extracted based on the method in §2.3 and stored for 7 days at 35°C in brown and clear vials was investigated. The results of the storage stability of these three pesticides in different pure solvents and extracted matrix solutions are shown in table 2.

**Table 2.** The stability of three pesticides in pure solvents and different extracted matrix solutions in brown and clear vials after storage at 35°C for 7 days (*n* = 3).

	brown vial			clear vial		
pesticide	solvent	peanut	soya bean	solvent	peanut	soya bean
hexane, recovery (%)						
phorate	98.52 ± 0.88	100.42 ± 3.02	98.63 ± 1.59	99.57 ± 2.76	94.44 ± 3.66	96.04 ± 2.65
disulfoton	97.66 ± 3.29	99.74 ± 0.37	99.04 ± 1.45	93.49 ± 1.16	67.30 ± 11.91	91.73 ± 3.19
fenthion	96.72 ± 7.08	101.7 ± 1.44	98.16 ± 3.43	98.08 ± 3.95	91.65 ± 6.43	94.69 ± 10.12
acetone, recovery (%)						
phorate	100.86 ± 2.40	85.59 ± 5.40	99.82 ± 1.38	94.89 ± 2.73	67.99 ± 5.17	77.78 ± 8.39
disulfoton	100.77 ± 3.67	79.07 ± 4.99	94.14 ± 5.11	95.56 ± 3.22	17.51 ± 1.78	55.97 ± 14.54
fenthion	99.68 ± 2.83	83.35 ± 5.46	97.6 ± 2.11	94.40 ± 7.76	93.91 ± 2.50	93.30 ± 5.30
ethyl acetate, recovery (%)						
phorate	—	63.62 ± 2.99	72.79 ± 1.79	—	29.27 ± 3.50	43.67 ± 3.20
disulfoton	—	13.19 ± 2.11	20.63 ± 0.89	—	0.00	2.44 ± 0.32
fenthion	—	70.81 ± 1.04	77.91 ± 3.09	—	41.96 ± 4.51	56.36 ± 1.76

When stored at 35°C for 7 days, the remaining of these three pesticide residues in all extracted matrix solutions was greater than 70% with the exception of phorate, disulfoton and fenthion in pure ethyl acetate and disulfoton in extracted matrix ethyl acetate solution. It was obvious that phorate, disulfoton and fenthion were not detected in the pure ethyl acetate solution after storage for 7 days. However the stability of the three pesticides was improved in the extracted matrix ethyl acetate solutions. Hence, it was stated that these three pesticides were degraded rapidly in pure ethyl acetate solvent, while the degradation rate was delayed in the extracted matrix ethyl acetate solution. Compared with the pure acetone solvent, the extracted matrix solution could increase the degradation of the three pesticides. The recovery of phorate and disulfoton were 95.56% and 94.89% respectively in pure solvent in clear vials, and the recoveries were 67.98% and 17.51% in extracted peanut solutions, and 77.78% and 55.97% in extracted soya bean solutions. For hexane, there were no differences between pure solvent and the extracted solutions.

It was concluded that pure solvents had a great influence on storage stability of pesticide residues according to the above results, and ethyl acetate was not conducive to the preservation of pesticides compared with acetone and hexane.

#### 3.2.2. Light

As shown in table 2, the stability improved when the sample was stored in brown vials than in clear vials especially in acetone or ethyl acetate extracted solutions. For hexane, the recovery of three pesticides had no obvious differences. For acetone extracted solution, the recoveries of phorate and disulfoton increased from 67.99 and 17.51% to 85.59 and 79.07 in peanut extracted solutions solvent, respectively. It was observed that the storage stability of disulfoton improved noticeably in brown vials. The same trend occurred in extracted soya bean matrix solutions. For ethyl acetate, these three pesticides were not detected in pure solvents. The recoveries of phorate, disulfoton and fenthion increased from 29.27%, 0.00% and 41.96% to 63.62%, 13.19% and 70.81% in peanut matrix solution, respectively. And the same trend occurred in extracted peanut solutions, the recoveries increased from 43.67%, 2.44% and 56.36% to 72.79%, 20.63% and 77.91% for phorate, disulfoton and fenthion, respectively. Disulfoton was easily degraded due to the presence of a thioether bond. Similar reports have shown that pesticides containing a thioether group were prone to degradation [17]. Therefore, ethyl acetate and acetone should be avoided as solvents when preparing standard solutions of organophosphorus pesticides containing sulfide bonds.

From the short discussion above, it could be demonstrated that light had a slight effect on storage stability [18]. For the special pesticides such as disulfoton studied, the light played an important effect on stability owing to the structure and the physico-chemical properties of disulfoton.

#### 3.2.3. Temperature

The present study confirmed the findings regarding the effect of temperature on storage stability of these three organophosphorus pesticides, as depicted in table 3. The recoveries of phorate, disulfoton and fenthion were 66.59%, 7.37% and 80.01% stored for 12 h at 4°C, and the recoveries increased to 87.38%, 40.52% and 95.04% when the temperature was reduced to −20°C. The recoveries of phorate, disulfoton and fenthion were 47.58%, 1.61% and 67.8% stored for 1 day at 4°C, and the recoveries were 74.96%, 23.09% and 87.98% stored for 1 day at −20°C in ethyl acetate. When stored for 3 days at 4°C, all of these three organophosphorus pesticides were less than the limit of quantitation. However, the recoveries were 59.33%, 3.67% and 64.93% at −20°C. The findings of this study concluded that even at −20°C, exposure to ethyl acetate resulted in 40.67%, 96.33% and 35.07% loss. It was obvious that the stability was significantly affected by temperature.

**Table 3.** The stability of three organophosphorus pesticides in ethyl acetate at 4°C and −20°C (*n* = 3).

	recovery (4°C)			recovery (−20°C)		
time	phorate	disulfoton	fenthion	phorate	disulfoton	fenthion
12 h	66.59 ± 3.49	7.37 ± 0.47	80.01 ± 2.86	87.38 ± 1.39	40.52 ± 1.24	95.04 ± 0.79
1 day	47.58 ± 3.05	1.61 ± 0.19	67.8 ± 1.27	74.96 ± 1.03	23.09 ± 0.46	87.98 ± 2.22
3 days	—	—	—	59.33 ± 1.13	3.67 ± 0.57	64.93 ± 1.12

## 4. Conclusion

The main conclusion that could be drawn was that the storage stability of these three organophosphorus pesticides, phorate, disulfoton and fenthion, was affected by type of solvent, light and temperature. This was an important finding in the understanding of the factors influencing storage stability. These three organophosphorus pesticides degraded quickly, especially in pure ethyl acetate solvent. In ethyl acetate, all three pesticides decreased greater than 30% when stored for 3 days at −20°C, while the results provided a basis that the stability could be improved in the matrix extracted solutions. We should pay more attention to the disulfoton in ethyl acetate. From the discussion above, it could be demonstrated that light had a slight effect on storage stability. For a special pesticide such as disulfoton, light played an important effect on stability due to the structure and the physico-chemical properties of disulfoton. In addition, these findings provided additional information that temperature could significantly affect the storage stability. It was concluded that even at −20°C, exposure to solvents or matrix extracted solution resulted in significant loss. Therefore, it could be assumed that the optimal storage conditions for these three organophosphorus pesticides of interest in peanut and soya bean include a lower temperature, storage in a brown vial and in acetone or hexane. In the future, new pesticides with similar structures could exhibit similar stability with the same matrix with reference to the results of this article.

## Data accessibility

This article does not contain any additional data.
